# The Influence of the Halide in the Crystal Structures of 1-(2,3,5,6-Tetrafluoro-4-pyridyl)-3-benzylimidazolium Halides

**DOI:** 10.3390/molecules27217634

**Published:** 2022-11-07

**Authors:** Udari A. I. Acharige, Graham C. Saunders

**Affiliations:** Te Aka Matua, School of Science, University of Waikato, Hamilton 3240, New Zealand

**Keywords:** imidazolium salt, anion–π interaction, π–π stacking, crystal structure, DFT calculation

## Abstract

The crystal structures of 1-(2,3,5,6-tetrafluoro-4-pyridyl)-3-benzylimidazolium chloride (**1**) and iodide (**3**) have been determined by single crystal X-ray diffraction. The crystal structure of **1** is similar to that of the bromide salt (**2**), possessing anion···C_5_F_5_N···C_6_H_5_ motifs, whilst that of **3** contains columns of alternating iodide anions and parallel tetrafluoropyridyl rings. All three crystal structures possess C(1)–H∙∙∙X^−^ and C(2)–H∙∙∙X^−^ hydrogen bonding. DFT calculations reveal that the strengths of the hydrogen bonding interactions lie in the order C(1)–H···X^−^ > C(3)–H···X^−^ > C(2)–H···X^−^ for the same halide (X^−^) and Cl^−^ > Br^−^ > I^−^ for each position. It is suggested that salt **3** adopts a different structure to salts **1** and **2** because of the larger size of iodide.

## 1. Introduction

The crystal structures of 1-polyfluoroaryl-3-benzylimidazolium bromide salts [1,2,3,4,5,6] have proved useful for studying a number of non-covalent interactions with importance in crystal engineering: charge-assisted hydrogen bonding [7,8], π–π stacking between polyfluoroaryl and aryl rings [9], lone pair–π interactions [10] and C–X∙∙∙Br^−^ halogen bonding [11]. It is evident from these studies that a number of interactions are common to all the crystal structures, but also that the nature of the cation has a large impact on which interactions control the crystal structure. For example, the crystal structures of 1-(4-halo-2,3,5,6-tetrafluorophenyl)-3-benzylimidazolium bromide are dependent on the halogen atom [6]: the structure of the salt of the chlorotetrafluorophenyl substituted cation contains columns of alternating polyfluoroarene and arene rings with π···π stacking interactions, the structure with the bromotetrafluorophenyl substituted cation contains columns of alternating bromide anions and polyfluoroarene rings with anion···π interactions, and the structure with the iodotetrafluorophenyl substituted cation contains columns of alternating iodine atoms and polyfluoroarene rings with lone pair···π interactions.

It is to be expected that polyatomic anions of different shapes and volumes, and which provide the possibility of multiple and different types of interactions with cations, would have a large influence on the crystal structure adopted by the salt. However, we hypothesize that simple monoatomic anions, which differ in size and consequent properties, such as polarizability, can also exert a strong influence on the crystal structures adopted by 1-polyfluoroaryl-3-benzylimidazolium salts. In order to test this hypothesis we chose to investigate the crystal structures of the halide salts of 1-(2,3,5,6-tetrafluoro-4-pyridyl)-3-benzylimidazolium (halide = chloride, **1**; bromide, **2**; iodide, **3**), the anions differing in size (the ionic radii are 1.67, 1.82 and 2.06 Å for chloride, bromide and iodide, respectively [12]) and polarizability (3.005, 4.168 and 6.294 Å^3^ [13]). In support, the nature of the halide is found to have a strong influence on the crystal structures of the chloride [14] and bromide [15] salts of 1,3-dibenzylimidazolium (CCDC codes: MOQBIE and WODHUT, respectively). Both crystallize as monohydrates in the monoclinic space group *P*2_1_/c, but with different packing arrangements: the chloride possessing C(1)–H···Cl^−^ and C(2)–H···Cl^−^ interactions and hydrogen-bonded [(Cl^−^)_2_.(H_2_O)_2_] rhomboids, the bromide possessing C(1)–H···Br^−^ and C(2)–H···OH_2_ interactions and chains of hydrogen-bonded alternating bromide anions and water molecules.

Here, we report the structures of 1-(2,3,5,6-tetrafluoro-4-pyridyl)-3-benzylimidazolium chloride (**1**) and iodide (**3**), and DFT calculations of the interactions present in these structures and that of the bromide salt (**2**) (CCDC reference: AMOCOV), which has been determined previously [3,16]. 
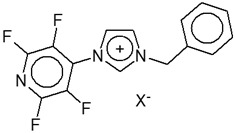
1 X = Cl, 2 X = Br, 3 X = I.

## 2. Results and Discussion

**Figure 1:**
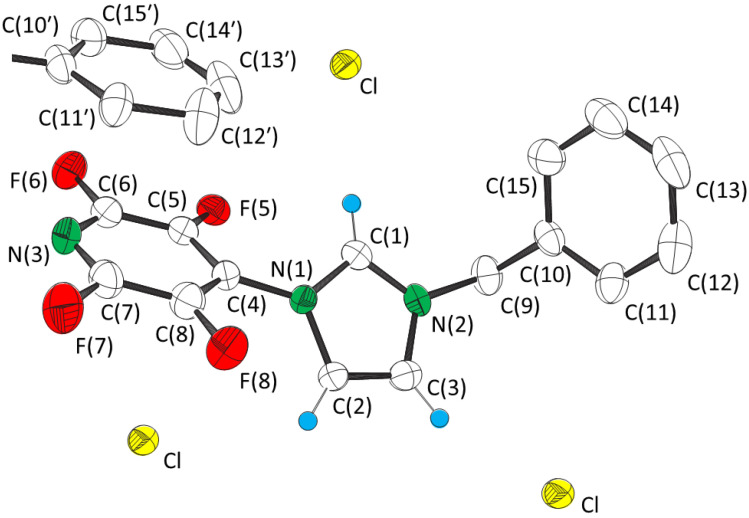
The structure of the cation of 1-(2,3,5,6-tetrafluoro-4-pyridyl)-3-benzylimidazolium chloride (**1**) indicating the positions of the three closest chloride anions and the phenyl ring of an adjacent cation. Thermal ellipsoids are at the 50% level. Hydrogen atoms not involved in hydrogen bonding are omitted for clarity.

**Figure 2:**
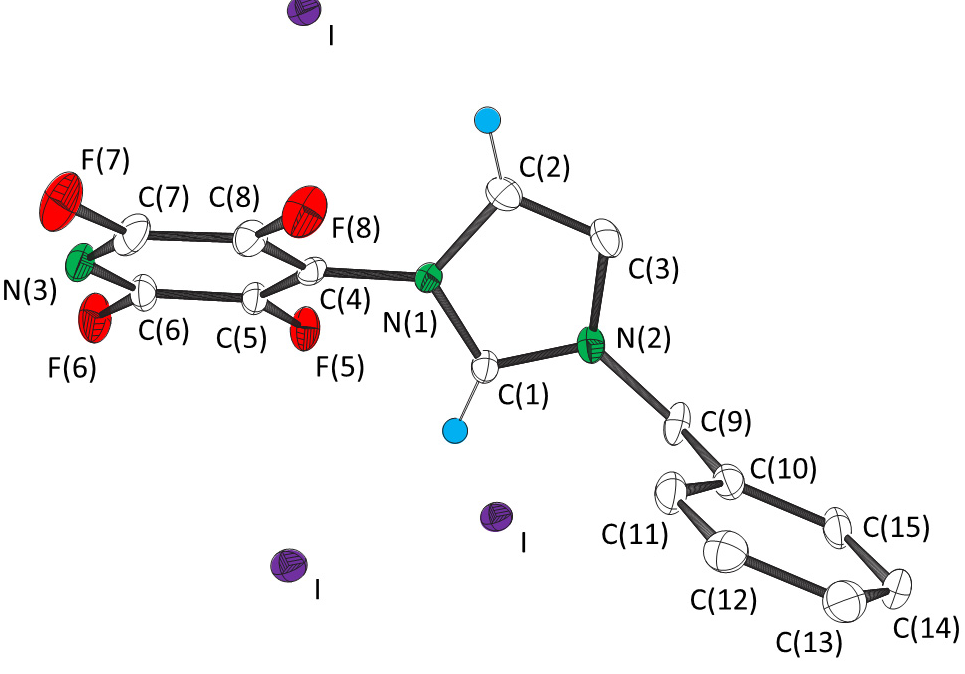
The structure the cation of 1-(2,3,5,6-tetrafluoro-4-pyridyl)-3-benzylimidazolium iodide (**3**) indicating the positions of the three closest iodide anions. Thermal ellipsoids are at the 50% level. Hydrogen atoms not involved in hydrogen bonding are omitted for clarity.

1-(2,3,5,6-Tetrafluoro-4-pyridyl)-3-benzylimidazolium chloride (**1**) and iodide (**3**) were prepared by treatment of the bromide salt (**2**) [3] with silver nitrate and then either tetramethylammonium chloride or sodium iodide. Anion metathesis was confirmed by the presence of [(NC_5_F_4_NC_3_H_3_NCH_2_Ph)_2_.X]^+^ and [(NC_5_F_4_NC_3_H_3_NCH_2_Ph).2X]^−^ peaks in the positive and negative ion mass spectra, respectively of **1** (X = Cl) and **3** (X = I), and the absence of [(NC_5_F_4_NC_3_H_3_NCH_2_Ph)_2_.Br]^+^ and [(NC_5_F_4_NC_3_H_3_NCH_2_Ph).2Br]^−^. Attempts to prepare the fluoride analogue by the same method were unsuccessful. Salt **1** crystallized from chloroform in the polar orthorhombic space group *P*na2_1_, the same as that of **2**. Salt **3** crystallized from dichloromethane in the centrosymmetric monoclinic space group *P*2_1_/c. Crystal data are given in Table 1 and selected distances and angles are given in Table 2. The structures of the cations of **1** and **3**, together with the positions of the closest halide anions, are shown in Figure 1 and Figure 2, respectively. To probe the relative importance of the interactions in the crystal structures DFT calculations were performed using the long-range corrected functional ωB97X-V [17] method and the basis set 6-311++G(2d,2p).

**Figure 3:**
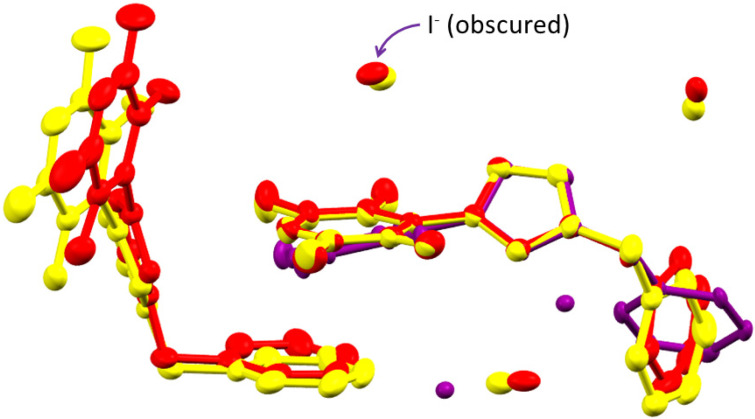
Overlay of the structures of the cations of 1-(2,3,5,6-tetrafluoro-4-pyridyl)-3-benzylimidazolium chloride (**1**) (yellow), bromide (**2**) (red) and iodide (**3**) (purple) with their three closest anions and cations involved in π···π stacking with the tetrafluoropyridyl ring. Thermal ellipsoids are at the 50% level. Hydrogen atoms are omitted for clarity.

The structure of the cation is virtually identical in the crystal structures of **1** and **2**, and that in the crystal structure of **3** (Figure 3) is similar. The bond distances and angles are similar to calculated values for the optimized structure of the isolated cation in the gas phase (Table 2). However, there are significant differences in the torsion angles between the cations of **1** and **3**, especially those with respect to the benzyl group. The largest difference is that the plane of the phenyl ring is perpendicular to N(2)–C(9) for **1** and **2**, but parallel for **3** (Figure 3).

All three structures possess charge assisted C(1)–H···X^−^ and C(2)–H···X^−^ hydrogen bonding, with that of salt **3** possessing two C(1)–H···X^−^ interactions. As expected, the C···X^−^ distances (Table 3) increase with the size of the anion. The C(1)···X^−^ distances are less than the sum of the van der Waal’s radius of carbon (1.70 Å) [18,19] and the ionic radius of the anion [12] (3.37, 3.52 and 3.76 Å for *r*_C_ + *r*_Cl_^−^, *r*_C_ + *r*_Br_^−^ and *r*_C_ + *r*_I_^−^, respectively), whilst C(2)···X^−^ is slightly longer. The angles around C(1) and C(2) (Table 2 and Table 3) are consistent with hydrogen bonding. The strengths of the C(1)–H···X^−^ interactions were calculated to be ca. −380 kJ mol^−1^ with the value decreasing slightly in the order **1** > **2** > **3** (Table 4). The values are ca. 10 kJ mol^−1^ weaker than the interactions with the halide ions in optimized positions. The interactions are 50 kJ mol^−1^ stronger for **1**, **2** and **3** than purely electrostatic interactions between the halide ion and the centre of positive charge, which is considered to be the midpoint of the N···N axis of the imidazolium ring [8]. The greater strength is a consequence of the hydrogen bonding, which for the interaction between neutral tetrafluoropyridylimidazole and anions was calculated to be ca. −50 kJ mol^−1^ for **1** and **2**, and −20 kJ mol^−1^ for the weaker C(1)–H···I^−^ interaction of **3**. The stronger C(1)–H···I^−^ interaction is augmented by an anion···π interaction. An energy of interaction of −51 kJ mol^−1^ was calculated for that between iodide and pentafluoropyridine with the same atom positions as those of the relevant experimentally determined ones of the cation of **3**.

The strengths of the C(2)–H···X^−^ interactions were calculated to be ca. 40 kJ mol^−1^ weaker than the respective C(1)–H···X^−^ interactions (Table 4), which is consistent with the longer C(2)···X^−^ distances (Table 3). The interactions are ca. 40 kJ mol^−1^ stronger than the purely electrostatic interactions between the halide ion and the centre of positive charge. All three interactions are augmented by anion···π interactions, with the interaction between tetrafluoropyridylimidazole and the anions calculated to be ca. −80 kJ mol^−1^ and that between pentafluoropyridine and the anions calculated to be ca. −55 kJ mol^−1^.

The structures of **1** and **2** also possess charge assisted C(3)–H···X^−^ hydrogen bonding. The C(3)···X^−^ distances are slightly longer than the sum of the van der Waal’s radius of carbon and the ionic radius of the anion, but shorter than the respective C(2)···X^−^ distances (Table 3). Although the anions are further from the centre of positive charge and there are no anion···π interactions, the C(3)–H···X^−^ interactions are stronger than the respective C(2)–H···X^−^ interactions. This is presumably a consequence of stronger hydrogen bonding arising from more preferable geometry; the anions are closer to the plane of the imidazolium ring and the difference between the N–C···X^−^ and C–C···X^−^ angles is smaller (30° cf. 55°). 

**Figure 4:**
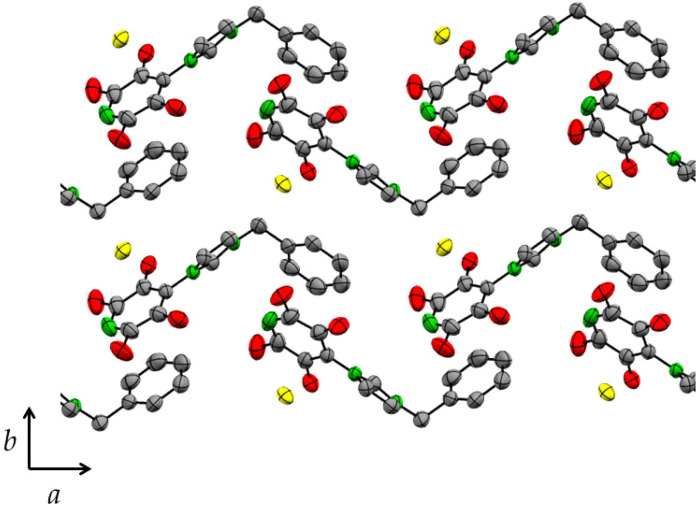
Crystal structure of 1-(2,3,5,6-tetrafluoro-4-pyridyl)-3-benzylimidazolium chloride (**1**) viewed parallel to the *c* axis. Thermal ellipsoids are at the 50% level. Hydrogen atoms are omitted for clarity.

The crystal structures of **1** (Figure 4) and **2** comprise chains of cations linked by tetrafluoropyridyl-phenyl π···π stacking interactions. The tetrafluoropyridyl and phenyl rings are almost parallel with a separation of ca. 3.3 Å. The energies of π···π stacking interactions between toluene and pentafluoropyridine with the same geometric parameters as the structures of **1** and **2** were calculated to both be −25 kJ mol^−1^. There is an anion···π interaction with the opposite face of the tetrafluoropyridyl ring to produce X^−^···C_5_F_4_N···C_6_H_5_ motifs. The chains are linked to form sheets parallel to the *a* and *b* axes by C(3)–H···X^−^ hydrogen bonding and anion···π interactions. The sheets are linked by C(1)–H···X^−^ hydrogen bonding almost parallel to the *c* axis (1.9° to the (100) plane, 21.1° to the (010) plane).

**Figure 5:**
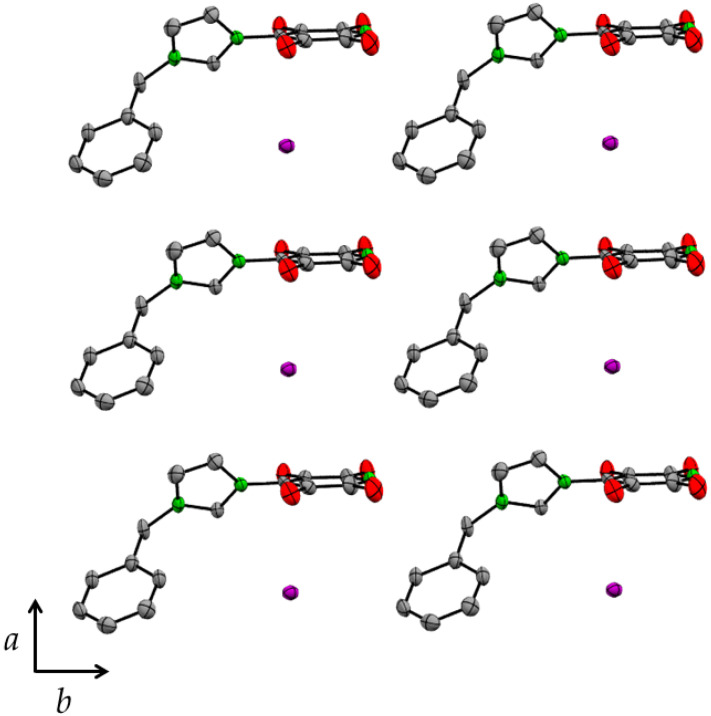
Crystal structure of 1-(2,3,5,6-tetrafluoro-4-pyridyl)-3-benzylimidazolium iodide (**3**) view parallel to the *c* axis. Thermal ellipsoids are at the 50% level. Hydrogen atoms are omitted for clarity.

The crystal structure of **3** comprises columns of alternating iodide anions and tetrafluoropyridyl rings parallel to the *a* axis (Figure 5). The iodide anions lie ca. 3.6 Å along the normal to the plane of the tetrafluoropyridyl ring from C(4) (Table 3). This distance is ca. 0.1 Å less than the sum of the van der Waals radius of carbon and the ionic radius of iodide. 

The DFT calculations (Table 4) reveal that the hydrogen bonding interactions lie in the order C(1)–H···X^−^ > C(3)–H···X^−^ > C(2)–H···X^−^ for the same halide and (X^−^) Cl^−^ > Br^−^ > I^−^ for each position. The former order is consistent with previous studies, and the latter consistent with anion size, and therefore H···X^−^ distance. The latter order is also displayed by the anion···π interactions, which are ca. −55 kJ mol^−1^. The π–π stacking interactions are about half the strength of the anion···π interactions. Although the structures of salts **1** and **2** possess a π–π stacking interaction in preference to the stronger anion···π interaction of the structure of salt **3**, the three C–H···X^−^ interactions are sufficiently strong to compensate for this. Consequently, the structure adopted by salts **1** and **2** is the more favoured. The calculated energies of the interactions between the cation of **2** and iodide at the same position as bromide are −360 and −330 kJ mol^−1^ for C(1)–H···I^−^ and C(3)–H···I^−^, respectively, which suggest that it is not the strength of the cation-anion interactions that determine which structure is adopted. It is therefore evident that salt **3** does not adopt this structure because of the larger size of iodide, which prevents the phenyl ring of the adjacent cation stacking with the tetrafluoropyridyl ring.

## 3. Materials and Methods

### 3.1. Instrumentation

The mass spectra were recorded on a Bruker Daltonics micrOTOF spectrometer.

### 3.2. Materials

Salt **2** [3] was prepared as described. Silver nitrate (Ajax Finechem), tetramethylammonium chloride (Sigma Aldrich) and sodium iodide (Reidel de Haën) were used as supplied. 

### 3.3. Preparation of 1-(2,3,5,6-Tetrafluoro-4-pyridyl)-3-benzylimidazolium Chloride (**1**) and Iodide (**3**)

Methanol (50 cm^3^) was added to a mixture of salt **2** (0.25 g, 0.64 mol) and silver nitrate (0.14 g, 0.82 mmol). After 15 min. the mixture was filtered twice through celite. The filtrate was divided into two equal fractions. To one fraction was added an excess of tetramethylammonium chloride, and the solution left for 1h., after which time it was filtered and the solvent removed by rotary evaporation to afford crude **1**. The product was dissolved in dichloromethane, filtered and the solvent removed by rotary evaporation to afford **1** as a colourless solid. The solvent was removed from the second fraction by rotary evaporation, and an excess of sodium iodide in acetone (25 cm^3^) added to the resulting solid. After 1h. the solution was filtered and the solvent removed by rotary evaporation to afford crude **3**. The product was dissolved in dichloromethane, filtered and the solvent removed by rotary evaporation to afford **3** as a pale yellow solid.

 **1.**  MS (positive ion): C_15_H_10_F_4_N_3_ requires 308.0811; found [M − Cl]^+^ 308.0653. C_30_H_20_F_8_N_6_^35^Cl requires 651.1310; found [2M − Cl]^+^ 651.0992. MS (negative ion): C_15_H_10_F_4_N_3_^35^Cl_2_ requires 378.0188; found [M + Cl]^−^ 378.0516. C_30_H_20_F_8_N_6_^35^Cl_3_ requires 723.0784; found [2M + Cl]^−^ 723.1200.
 **3.**  MS (positive ion): C_15_H_10_F_4_N_3_ requires 308.0811; found [M − I]^+^ 308.1033. C_30_H_20_F_8_N_6_I requires 743.0666; found [2M − I]^+^ 743.1140. MS (negative ion): I requires 126.9045; found [M − C_15_H_10_F_4_N_3_]^−^ 126.9181. C_15_H_10_F_4_N_3_I_2_ requires 561.8900; found [M + I]^−^ 561.9393.

### 3.4. X-ray Crystallography

Crystals of **1** and **3** were obtained by slow evaporation of solvent from solutions in chloroform and dichloromethane, respectively. Crystal data are listed in Table 1. Diffraction data were collected on an Agilent SuperNova, single source at offset, Atlas diffractometer with graphite-monochromated Cu—K_α_ radiation. The structures of **1** and **3** were solved using Olex2 [20] and refined with the olex2.refine [21] refinement package using Gauss-Newton minimization. The non-hydrogen atoms were refined with anisotropic thermal parameters. Hydrogen atom positions were added in idealized positions and a riding model with fixed thermal parameters (Uij = 1.2 Ueq for the atom to which they are bonded (1.5 for CH_3_)) was used for subsequent refinements. The function minimized was [w(|*F*_o_|^2^ − |*F*_c_|^2^)] with reflection weights *w*^−1^ = [σ^2^ |*F*_o_|^2^ + (g1*P*)^2^ + (g2*P*)] where *p* = [max |*F*_o_|^2^ + 2|*F*_c_|^2^]/3. 

CCDC 2063365 (**1**) and 2063364 (**3**) contain the supplementary crystallographic data for this paper. These data can be obtained free of charge from The Cambridge Crystallographic Data Centre via www.ccdc.cam.ac.uk/data_request/cif.

### 3.5. Density Functional Theory Calculations

DFT calculations were performed using Q-CHEM [22] with the long-range corrected functional ωB97X-V [17] method with the basis set 6-311++G(2d,2p). The energies of interaction were calculated as the difference between the energy of the species and the sum of those of the component ions and molecules corrected for basis set superposition error (BSSE) [23]. 

A neutron diffraction study has revealed that all the C–H bond distances of the cation of 1-(2,3,5,6-tetrafluoropyridyl)-3-benzylimidazolium bromide are 1.083 Å within experimental error [3]. Consequently C–H bonds of the experimental structures were normalized to 1.083 Å before calculation of their energies and optimization of the positions of the halide ions. Calculations performed on model systems involving tetrafluoropyridylimidazole and pentafluoropyridine used the positions of the relevant atoms of the experimentally determined salts with the *para* C–F bond distance fixed at 1.322 Å [24].

## 4. Conclusions

1-(2,3,5,6-Tetrafluoro-4-pyridyl)-3-benzylimidazolium chloride and bromide adopt similar crystal structures with X^−^···C_5_F_4_N···C_6_H_5_ motifs, whilst the iodide salt, because of the larger size of the anion, adopts a different crystal structure containing columns of alternating tetrafluoropyridyl rings and iodide anions. The strength of charge-assisted hydrogen bonding interactions lie in the order C(1)–H···X^−^ > C(3)–H···X^−^ > C(2)–H···X^−^ for the same anion and Cl^−^ > Br^−^ > I^−^ for each position. The strengths of the X^−^···C_5_F_4_N interactions also decreases in the order Cl^−^ > Br^−^ > I^−^.

## Figures and Tables

**Table 1:** Crystallographic data for 1-(2,3,5,6-tetrafluoropyridyl)-3-benzylimidazolium chloride (**1**) and iodide (**3**) ^1^.

	1	3
Formula	C_15_H_10_ClF_4_N_3_	C_15_H_10_IF_4_N_3_
Formula weight	343.71	435.16
Crystal system	orthorhombic	monoclinic
Space group	*P*na2_1_	*P*2_1_/c
*a*, Å	13.3971(6)	7.24395(17)
*b*, Å	8.8393(4)	10.4696(3)
*c*, Å	12.5132(5)	20.5728(5)
*β*, ^o^	90	93.535(2)
*V*, Å^3^	1481.83(11)	1557.30(7)
*Z*	4	4
*D*_c_ (g cm^−3^)	1.541	1.856
Crystal size (mm^3^)	0.126 × 0.075 × 0.024	0.201 × 0.084 × 0.063
*μ* (mm^−1^)	2.728	16.587
*θ* range (°)	3.00→74.19	4.31→73.90
Total reflections	4568	9013
Unique reflections (*R*_int_)	2400(0.0269)	3056(0.0293)
Observed reflections [*I* > 2 (*I*)]	2155	2796
Parameters	208	208
Final *R* indices [*I* > 2*σ*(*I*)]	*R*_1_ = 0.0394,	*R*_1_ = 0.0240,
	*wR*_2_ = 0.1173	*wR*_2_ = 0.0600
*R* indices (all data)	*R*_1_ = 0.0493,	*R*_1_ = 0.0281,
	*wR*_2_ = 0.1350	*wR*_2_ = 0.0629
Weighting scheme	*w* = 1/[*σ*^2^(*F*_o_)^2^ + {0.1002 (*F*_o_^2^ + 2*F*_c_^2^)/3}^2^]	*w* = 1/[*σ*^2^(*F*_o_)^2^ + {0.0352 (*F*_o_^2^ + 2*F*_c_^2^)/3}^2^ + 0.5871(*F*_o_^2^ + 2*F*_c_^2^)/3]
Max., min. Δ*ρ* (eÅ^−3^)	0.396, −0.340	0.566, −0.773
Goodness of fit on *F*^2^	1.055	1.054
Flack parameter	−0.03(2)	―

^1^ Estimated standard deviations are given in parentheses. Data were collected at 100(1) K with graphite monochromated radiation (λ = 1.54184 Å).

**Table 2:** Selected experimental and calculated bond distances (Å) and angles (°) for 1-(2,3,5,6-tetrafluoropyridyl)-3-benzylimidazolium chloride (**1**), bromide (**2**), and iodide (**3**) ^1^.

	1	2 [2]	3	Calc ^2^
C(1)–N(1)	1.337(6)	1.342(2)	1.346(3)	1.341
C(1)–N(2)	1.323(5)	1.313(2)	1.324(4)	1.322
N(1)–C(2)	1.397(4)	1.393(2)	1.391(3)	1.389
N(1)–C(4)	1.406(5)	1.416(2)	1.413(3)	1.423
N(2)–C(3)	1.385(4)	1.385(2)	1.380(4)	1.383
N(2)–C(9)	1.485(5)	1.485(2)	1.469(3)	1.489
C(2)–C(3)	1.340(6)	1.348(3)	1.345(4)	1.353
C(9)–C(10)	1.498(5)	1.511(3)	1.512(4)	1.506
N(1)–C(1)–N(2)	107.7(3)	107.7(2)	107.9(2)	108.6
C(1)–N(1)–C(2)	108.6(3)	109.2(2)	108.9(2)	108.5
N(1)–C(2)–C(3)	106.9(3)	106.2(2)	106.3(3)	106.7
N(2)–C(3)–C(2)	107.0(3)	107.1(2)	107.8(2)	107.3
C(1)–N(2)–C(3)	109.8(3)	109.9(2)	109.2(2)	108.9
C(1)–N(1)–C(4)	125.7(3)	125.1(2)	124.4(2)	125.3
C(1)–N(2)–C(9)	125.9(3)	125.4(2)	124.2(2)	125.5
N(2)–C(9)–C(10)	112.8(3)	110.7(2)	111.9(2)	111.1
∠ C_5_F_4_N^plane^ C_3_N_2_^plane 3^	42.1(5)	39.4(3)	52.3(5)	47.0
∠ C_6_H_5_^plane^ C_3_N_2_^plane 3^	67.1(5)	72.3(3)	86.5(5)	89.3
∠ C_5_F_4_N^plane^ C_6_H_5_^plane 3^	89.3(5)	89.8(3)	39.0(5)	50.2
C(1)–N(1)–C(4)–C(5)	40.2(5)	−144.3(2)	−51.8(4)	46.9
C(1)–N(1)–C(4)–C(8)	−143.7(4)	39.7(2)	125.7(3)	−133.6
C(1)–N(2)–C(9)–C(10)	84.5(4)	98.1(2)	−97.3(3)	140.0
C(3)–N(2)–C(9)–C(10)	−95.8(4)	−80.2(2)	76.2(3)	−41.8
N(2)–C(9)–C(10)–C(11)	92.9(4)	81.7(2)	23.2(3)	106.6
N(2)–C(9)–C(10)–C(15)	−87.0(4)	−96.1(2)	−157.1(2)	−72.6

^1^ Estimated standard deviations are given in parentheses. ^2^ Data for the cation structure optimized using the ωB97X-V method and the 6-311++G(2d,2p) basis set. ^3^ C_5_F_4_N^plane^ and C_6_H_5_^plane^ represent the planes defined by the six atoms of the tetrafluoropyridyl and phenyl rings, respectively. C_3_N_2_^plane^ represents the plane defined by the three carbon and two nitrogen atoms of the imidazolium ring.

**Table 3:** This Selected experimental and calculated interionic distances (Å) and angles of (°) of 1-(2,3,5,6-tetrafluoropyridyl)-3-benzylimidazolium chloride (**1**), bromide (**2**), and iodide, (**3**) ^1^.

	1 (X = Cl)		2 (X = Br)		3 (X = I)	
	Expt	Calc ^2^	Expt	Calc ^2^	Expt	Calc ^2^
C(1)···X^−^	3.356(4)	3.033	3.467(2)	3.169	3.576(3) 3.705(3)	3.198 3.351
N(1)–C(1)···X^−^	125.6(2)	140.9	135.0(1)	132.6	93.3(2) 136.9(2)	91.2 144.2
N(2)–C(1)···X^−^	126.2(2)	110.2	117.3(1)	119.7	136.2(2) 97.8(2)	127.3 106.7
C_3_N_2_^plane^···X^− 3^	0.357(4)	0.489)	0.15(1)	0.10	2.288(3) 2.130(3)	2.427 0.438
C_5_F_4_N^plane^···X^− 3^	–	–	–	–	3.596(4)	3.357
C_5_F_4_N^+^···X^− 4^	–	–	–	–	3.815(4)	3.836
C(4)···X^−^	–	–	–	–	3.604(3)	3.414
C(2)···X^−^	3.499(4)	2.917	3.834(2)	3.069	3.973(3)	3.367
N(1)–C(2)···X^−^	87.0(2)	82.2	83.66(9)	82.7	84.1(2)	85.8
C(3)–C(2)···X^−^	141.0(3)	120.8	141.0(1)	122.1	138.9(2)	139.5
C_3_N_2_^plane^···X^− 3^	2.218(4)	2.518	2.388(2)	2.605	2.577(3)	2.154
C_5_F_4_N^plane^···X^− 3^	3.192(4)	2.967	3.315(2)	3.111	3.631(4)	3.285
C_5_F_4_N^+^···X^− 4^	3.372(4)	3.518	3.414(2)	3.599	3.847(4)	3.634
C(4)···X^−^	3.289(4)	3.024	3.441(2)	3.146	3.671(3)	3.292
C(3)··· X^−^	3.449(4)	3.134	3.593(2)	3.285	–	–
N(2)–C(3)···X^−^	110.3(2)	101.3	109.7(1)	101.4	–	–
C(2)–C(3)···X^−^	142.6(3)	150.9	139.6(1)	145.6	–	–
C_3_N_2_^plane^···X^− 3^	0.140(4)	0.358	0.97(1)	1.061	–	–
C(3)··· X^−^	3.449(4)	3.134	3.593(2)	3.285	–	–
∠ C_6_H_5_^plane^ C_5_F_4_N ^plane 3^	2.6(3)	–	4.8(2)	–	–	–
C_6_H_5_^+^···C_5_F_4_N ^plane 3^	3.322(6)	–	3.271(3)	–	–	–
C_6_H_5_^plane^···C_5_F_4_N^+ 3,4^	3.351(6)	–	3.374(3)	–	–	–
C_6_H_5_^+^···C_5_F_4_N^+ 4^	3.499(6)	–	3.614(3)	–	–	–
C_5_F_4_N ^plane^···C_5_F_4_N ^plane 3,5^	–	–	–	–	7.244(4)	–
I^−^···I^− 6^	–	–	–	–	7.2439(3)	–
I^−^··· C_5_F_4_N^+^···I^− 7^	–	–	–	–	142.0(1)	–
∠ column C_5_F_4_N ^plane 8^	–	–	–	–	86.0(2)	–

^1^ Estimated standard deviations are given in parentheses. ^2^ Data for the optimized positions of the halide anion relative to the experimentally determined structure of the cation with C–H bond distances normalized to 1.083 Å. Calculations were performed using the ωB97X-V method and the 6-311G++(2d,2p) basis set. ^3^ C_5_F_4_N^plane^ and C_6_H_5_^plane^ represent the planes defined by the six atoms of the tetrafluoropyridyl and phenyl rings, respectively. C_3_N_2_^plane^ represents the plane defined by the three carbon and two nitrogen atoms of the imidazolium ring. ^4^ C_5_F_4_N^+^ and C_6_H_5_^+^ represent the centroids of the rings defined by the six atoms of the tetrafluoropyridyl and phenyl rings, respectively. ^5^ The separation between the planes of the rings within a column. ^6^ The separation between the iodide anions within a column. ^7^ C_5_F_4_N^+^···I^−^··· C_5_F_4_N^+^ has the same value as I^−^··· C_5_F_4_N^+^···I^−^. ^8^ The angle subtended by the column and the plane defined by the six atoms of the tetrafluoropyridyl ring.

**Table 4:** Calculated energies of interaction (kJ mol^−1^) between the halide anion at different positions and the cation of 1-(2,3,5,6-tetrafluoropyridyl)-3-benzylimidazolium chloride **1**, bromide, **2**, and iodide, **3**, and related model systems ^1^.

Halide Close to:	Salt	Experimental Structure	Optimized Halide Position ^2^	Electrostatic Interaction (r, Å) ^3^	Tetrafluoropyridylimidazole ^4^	Pentafluoropyridine ^4,5^
C(1)	**1**	−392	−406	−336 (4.138)	−65	
	**2**	−374	−380	−327 (4.244)	−47	
	**3**	−366 −342	−371 −354	−336 (4.134) −322 (4.318)	−68 −20	−51
C(2)	**1**	−352	−369	−311 (4.466)	−86	−62
	**2**	−332	−351	−295 (4.702)	−77	−56
	**3**	−324	−334	−290 (4.790)	−71	−51
C(3)	**1**	−362	−379	−287 (4.832)	−31	
	**2**	−343	−353	−281 (4.938)	−22	

^1^ Calculations with correction for BSSE were performed using the ωB97X-V method and the basis set 6-311++G(2d,2p). The C–H bond distances were normalized to 1.083 Å. ^2^ Data for the optimized positions of the halide anion relative to the experimentally determined structure of the cation. ^3^ The energy of interaction (e^2^/4πε_0_r) between point charges located at the centre of the anion and at the midpoint of the two nitrogen atoms of the imidazolium ring. The distance between the two points is given in parentheses. ^4^ Using the experimentally determined positions of the relevant atoms. ^5^ The fluorine atom in the 4-position of pentafluoropyridine positioned to give a C–F bond distance of 1.322 Å with C–C–F angles identical to the C–C–N angles of the cation.

## Data Availability

Copies of the data are available from the authors.

## Sample Availability

Samples of the compounds are not available from the authors.
